# Benchmarking supervised classifiers within a design-of-experiments framework: Robust statistical inference in text mining

**DOI:** 10.1016/j.mex.2026.104060

**Published:** 2026-07-24

**Authors:** Angelo Maria Sabatini

**Affiliations:** The BioRobotics Institute, Scuola Superiore Sant’Anna, Pisa, Italy

**Keywords:** Text mining, Supervised classification, Design of experiments, Statistical inference, Data leakage

## Abstract

Supervised classification based on Bag-of-Words representations is widely used in literary text mining, yet benchmarking practices often remain methodologically fragile. Common problems include feature selection before train/test separation, comparisons based on non-shared resampling splits, and inferential conclusions drawn from average accuracy alone. These choices may produce misleading significance and overstate marginal performance differences between classifiers.

This article presents a reproducible workflow for benchmarking supervised classifiers within a design-of-experiments framework for text analysis in R, using Dante’s Divina Commedia as an empirical testbed. The workflow combines leakage-free preprocessing, shared Monte Carlo train/test splits, paired statistical comparison, pilot-based power assessment, and validation through deliberately mispaired designs. Elastic-net multinomial logistic regression and linear support vector machine are used as competing classifiers. The objective is not to propose a new classifier, but to show how established methods can be applied under statistically coherent conditions.

Shared train/test splits should be treated as part of the inferential design rather than as a technical detail of model fitting.

Leakage-free preprocessing remains essential even when bias appears numerically small, because leakage can alter classifier ranking and distort inferential interpretation.

Small performance differences should be interpreted alongside model transparency, inferential stability, and practical relevance, rather than through accuracy alone.

## Graphical abstract

**Figure:**
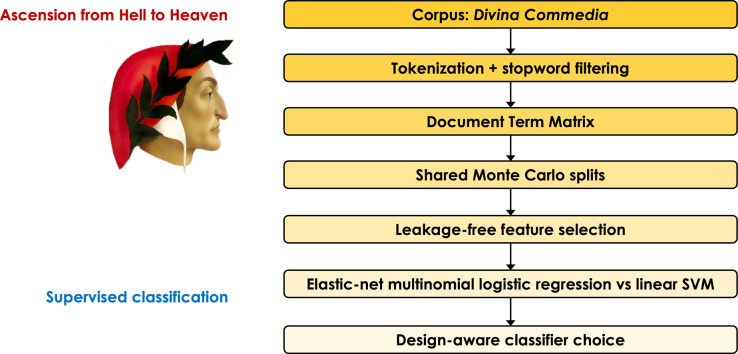

## Specifications table

**Table:** 

**Subject area**	Computer Science
**More specific subject area**	Computation and language; Machine learning; Statistical benchmarking
**Name of your method**	Benchmarking supervised classifiers within a design-of-experiments framework
**Name and reference of original method**	1. Kuhn, M., Silge, J. (2023). Tidy modeling with R. O’Reilly Media. 2. Hvitfeldt, E., Silge, J. (2021). Supervised machine learning for text analysis in R. CRC Press. 3. Oehlert, G.W. (2000). A first course in design and analysis of experiments. W.H. Freeman & Co.
**Resource availability**	The complete reproducible workflow is available at https://github.com/angelosabatini/repo_MethodsX and is permanently archived on Zenodo (doi:10.5281/zenodo.21534964).

## Background

Supervised machine learning is routinely used for text analysis in Digital Humanities (DH) and Computational Literary Studies (CLS) [1,2]. At the core of many such applications lies the Bag-of-Words (BoW) model, a simplifying representation in which a text is reduced to a multiset of its words, preserving word multiplicity while disregarding grammar, syntax, and word order [3].

In technical terms, BoW is a feature extraction method that maps each document into a high-dimensional vector space. A corpus is first used to define a vocabulary of *N* unique tokens. Each document is then represented as a numerical vector **v** of length *N*, where each element *v_i_* corresponds either to the count (term frequency) or to the presence (binary encoding) of the *i* th token in the vocabulary. This is one of the most common ways to represent textual information in a corpus, although alternative weighting schemes and feature representations are also possible [4].

This representation reduces text to a sparse vector that can be directly used as input for supervised classification models. When multiple documents are considered together, these vectors are naturally organized into a Document-Term Matrix (DTM), where rows correspond to statistical units of analysis (documents) and columns correspond to terms in the vocabulary.

In literary applications, rows often represent novels, poems, chapters, sonnets, cantos or other interpretable textual units, while columns define the lexical feature space used for classification. This matrix representation is not merely a computational convenience: it determines how metadata, class labels, and document boundaries enter the analysis, and therefore directly affects the validity of train/test separation, feature selection, and statistical comparison between competing classifiers.

Supervised machine learning workflows based on DTM and discriminative classifiers such as elastic-net multinomial logistic regression (EN-MNLR) and linear support vector machine (SVM) are now routinely applied in text mining and computational literary studies [5]. These approaches are supported by mature software ecosystems and well-established methodological references, particularly within the R environment, where packages such as tidytext, glmnet, kernlab, and the tidymodels framework facilitate transparent preprocessing, model tuning, and reproducible statistical evaluation [6].

The present work is implemented in R. However, the methodological validity of the proposed workflow does not depend on a specific programming language: the same design principles apply equally to equivalent implementations in Python or other machine learning environments.

Despite this maturity, practical applications often suffer from avoidable weaknesses: feature selection performed before train/test separation, non-shared train/test splits across competing classifiers, and comparisons based only on average accuracy [5,6].

These issues are not merely technical details. Small performance differences may become statistically misleading, and marginal gains in predictive accuracy may be incorrectly preferred over more transparent and interpretable models. This article proposes a design-of-experiments framework for benchmarking supervised classifiers in text mining, emphasizing leakage-free preprocessing, shared Monte Carlo splits, paired comparison, and public release of reproducible code.

## Method details

### Corpus definition and document structure

The workflow is illustrated using the *Divina Commedia* as an empirical testbed for supervised text classification. The *Divina Commedia* – the major narrative poem of Dante Alighieri (1265–1321) – describes the imagined journey of the poet through the afterlife, from sin to beatitude, and is divided into three cantiche – *Inferno* (Hell), *Purgatorio* (Purgatory), and *Parad*iso(Heaven) – reflecting the theological structure of medieval Christianity. Each cantica is composed of multiple cantos (34, 33 and 33), which provide naturally interpretable document-level units for classification [[7], [8], [9]].

Two classification settings are considered. The first is a binary task contrasting *Inferno* and *Paradiso*, representing the easiest discriminative scenario and serving as a sanity check for the workflow. The second is a ternary task including *Purgatorio* as an intermediate class, which introduces greater ambiguity and reflects a more realistic benchmarking problem. The inclusion of *Purgatorio* is methodologically relevant because it occupies a structurally transitional position between the lexical and thematic poles of the other two cantiche [10].

The literary corpus is not used here as an object of philological interpretation, but as a transparent and replicable benchmark for evaluating supervised classifiers under controlled experimental conditions. The focus of the study is therefore methodological rather than literary: the aim is to assess how experimental design influences classifier comparison and model selection.

### Text preprocessing and tokenization

Text preprocessing follows a BoW workflow implemented in R, but tokenization is not treated as a language-agnostic default operation. In literary corpora, and particularly in Dante’s historical Italian vernacular (*volgare*), token identification depends on explicit linguistic choices that directly affect the resulting feature space [9,11].

Each canto is tokenized at the word level after basic normalization of the text, including case standardization and punctuation removal. Particular attention is given to the treatment of apostrophes, which play a structurally relevant role in Italian and are especially frequent in Dante’s *volgare*. For this reason, tokenization is performed through a custom rule-based procedure designed to preserve linguistically meaningful units rather than relying solely on default package settings.

No stemming or lemmatization is applied, in order to preserve lexical transparency and maintain direct interpretability of the resulting features. This choice is particularly important in literary applications, where morphological variation may itself carry stylistic and interpretative relevance [12,13]. Stopword filtering is applied to reduce the influence of high-frequency functional words with limited discriminative value for document classification. Rather than adopting a generic modern Italian stopword list, stopwords are calibrated specifically for the Dantean corpus and conservatively selected among the most frequent and widely distributed tokens across cantos, followed by manual refinement to avoid removing lexically meaningful forms [9]. The stopword list is intentionally conservative to preserve lexical transparency and also facilitate comparison with previous studies using similar literary corpora. In addition, tokens shorter than three characters are excluded, reducing the impact of isolated articles, prepositions, and other very short functional forms that contribute little to discriminative modeling while increasing sparsity and noise.

After preprocessing, the corpus consisted of 100 canto-level documents and 101,604 retained tokens, with a final vocabulary of 500 highest-frequency lexical features selected for DTM construction, in line with common stylometric practice for medium-sized literary corpora [7]. The custom stopword list included 73 high-frequency functional terms. These were manually refined to preserve lexical interpretability while reducing non-informative variation.

These preprocessing steps define the lexical feature space used to construct the DTM, which serves as the common input for the supervised classifiers. Since tokenization and DTM construction are global operations, any feature selection step that depends on frequency ranking or discriminative relevance needs to be performed strictly within the training partitions only, in order to prevent information leakage from the test set. Leakage occurs whenever information from the held-out evaluation data indirectly influences model construction, producing overly optimistic estimates of predictive performance [6].

The numerical impact of this contamination may sometimes appear small, especially in stable classification settings, but its methodological consequences remain substantial: once train and test information are mixed, inferential comparison between competing models is no longer strictly valid. For this reason, leakage-free preprocessing is treated here as a design requirement rather than a technical refinement. This distinction is central to the workflow and is made explicit in the subsequent sections.

### Document-term matrix construction

After preprocessing, the retained tokens are organized into the DTM. It is worth noting that the analysis deliberately focuses on count-based lexical representations rather than semantic embeddings or transformer-based approaches [5]. The objective of the paper is not to maximize predictive performance, but to provide a statistically transparent workflow for classifier evaluation.

Count-based DTM representations remain particularly suitable for this purpose because they preserve interpretability, allow direct inspection of discriminative terms, and integrate naturally with classical inferential procedures. In literary text analysis, the possibility of moving from predictive classification to interpretative reflection depends not only on the feature representation, but also on the type of classifier adopted [14,15]. EN-MNLR and SVM differ substantially in their interpretability, yet both are considered here within the same methodological framework. Model selection often requires balancing predictive performance against transparency, robustness, and explanatory value, rather than simply maximizing accuracy.

### Shared Monte Carlo train/test splits

Repeated Monte Carlo train/test splits are generated and shared across all classifiers. In each repetition, the same partition of documents is used for both EN-MNLR and SVM, allowing direct paired comparison of predictive performance. Operationally, this is achieved by fixing and storing the random seeds used to initialize the resampling procedure, so that identical train/test partitions are reproduced across competing models [16].

This design avoids a common benchmarking weakness in which different classifiers are evaluated on different random splits and subsequently compared using independent tests. When shared splits are used, the performance difference between models becomes a paired quantity, substantially improving inferential validity and statistical power for the same number of train/test repetitions [17].

This distinction is particularly important because the effective amount of inferential information depends not only on the number of Monte Carlo repetitions, but also on whether repeated evaluations are structurally paired. Shared splits allow stronger statistical comparison without increasing the total computational budget, while non-shared splits must be treated as independent samples. Treating non-shared repetitions as if they were paired observations is a common but methodologically incorrect practice that can produce unstable or misleading inferential conclusions.

### Leakage-free feature selection

In the present workflow, tokenization is performed once at the corpus preparation stage and does not enter the resampling workflow, since the custom tokenizer relies on fixed linguistic rules rather than corpus-dependent estimation [5]. For this reason, token identification does not depend on train/test partitioning. By contrast, all preprocessing steps involving feature ranking, filtering, or discriminative selection are implemented within each split, ensuring that they are estimated exclusively on the training data and then consistently applied to the corresponding held-out test sets.

Feature engineering is itself part of the inferential design, not merely a preprocessing convenience. A leakage-free implementation is therefore essential to the modeling process and is well aligned with the good practices supported by the recipes component of the tidymodels ecosystem in R [6]. Selecting features on the complete corpus allows information from the future test set to influence model construction, producing a classical form of data leakage. Although the resulting bias may appear numerically small, it can substantially alter inferential comparisons, especially when performance differences between classifiers are marginal.

A parallel leaky implementation is retained only for validation purposes, allowing direct empirical comparison between correct and incorrect design choices.

### Classifier specification and hyperparameter tuning

EN-MNLR is implemented through the glmnet framework [18], with hyperparameter tuning focused on the elastic-net mixing parameter and the regularization penalty, selected through internal resampling procedures. Its coefficient structure preserves a direct and interpretable relation between lexical features and classification outcomes, making interpretative inspection possible beyond predictive performance alone.

SVM is implemented through the kernlab framework [19] and included as a strong baseline classifier commonly used in text classification tasks. Its tuning focuses primarily on the cost parameter, using the same internal resampling logic adopted for EN-MNLR. Since SVM generally offers reduced interpretability compared to regularized regression models, it provides an important contrast between predictive strength and model transparency.

The comparison is intentionally restricted to these two models. The objective is not exhaustive classifier ranking, but the evaluation of inferential design under realistic and interpretable benchmarking conditions.

### Performance metrics and paired statistical comparison

Model performance is evaluated using four complementary metrics: accuracy, balanced accuracy, Matthews Correlation Coefficient (MCC), and macro-averaged F1 score [8]. Accuracy provides an overall summary of classification success, while balanced accuracy provides a class-wise performance measure that remains informative under class imbalance. MCC is included because it offers a robust summary of multiclass agreement and is less sensitive to marginal distribution distortions [20]. Macro-F1 provides an additional class-balanced perspective focused on precision and recall symmetry.

Because these performance measures are bounded sample statistics and often exhibit asymmetric or left-skewed empirical distributions, especially when classification performance is relatively high, strict normality assumptions are not always reliable. Variance homogeneity may also be unstable across classifiers, particularly under independent comparisons. For this reason, statistical inference is conducted within a non-parametric framework using paired Wilcoxon signed-rank tests.

Given that classifiers are evaluated on shared Monte Carlo splits, model comparison is performed using paired Wilcoxon procedures to evaluate whether observed differences between EN-MNLR and SVM remain statistically meaningful once split variability is properly controlled.

Preliminary power assessment during workflow piloting is nevertheless conducted using standard parametric approximations [21], as a practical way to evaluate the expected sensitivity of the design before full resampling analysis. This step is inexpensive, rarely reported explicitly, and helps prevent overinterpretation of small but unstable performance differences.

When statistical benchmarking involves a larger set of competing models, the same logic naturally extends to ANOVA designs, where “classifier” becomes a within-subjects factor under shared splits, or a between-subjects factor when independent splits are used. In such cases, one-way ANOVAs (or their non-parametric equivalents) and appropriately controlled post-hoc comparisons become necessary to preserve inferential validity [17].

For methodological illustration, two additional contrasts are also examined: independent comparisons based on non-shared splits and Mann-Whitney U tests, and deliberately mispaired comparisons in which non-shared repetitions are incorrectly treated as paired observations. Independent comparisons were obtained by drawing non-overlapping subsets of repetitions from the same Monte Carlo reservoir of train/test splits. Because each split was generated independently through pseudo-random resampling, these non-shared subsets can be treated as independent benchmarking samples. This allows paired and independent inferential designs to be compared without changing the underlying corpus, preprocessing workflow, or feature space. These additional scenarios reproduce common benchmarking mistakes and help quantify their practical inferential consequences.

### Reproducibility infrastructure

The full workflow is organized as a reproducible research pipeline implemented in R. All scripts used for preprocessing, split generation, model fitting, hyperparameter tuning, metric computation, figure production, and statistical comparison are released and fully available in a structured GitHub repository.

The repository includes processed datasets, predefined shared train/test splits, model outputs, summary tables, and graphical outputs, allowing the complete analysis to be fully reproduced. Tokenized outputs are provided directly, while the custom tokenizer itself is not distributed, since tokens are fixed upstream and methodological reproducibility depends on the resulting analytical objects rather than on repeated raw text preprocessing. The full primary text of the corpus is not redistributed because it is publicly available from standard digital sources and is not required for methodological reproducibility.

The workflow is intentionally designed around transferable analytical objects rather than a single immutable corpus. Alternative editions or translations of the *Divina Commedia*, or even other textual corpora can be analyzed using the same pipeline provided that documents are appropriately organized to produce the DTM. This makes the method reusable beyond the specific case study presented here.

## Method validation

Table 1 summarizes the full validation architecture of the proposed workflow, including classification tasks, preprocessing conditions, Monte Carlo resampling strategy, internal tuning procedures, and the inferential design used for classifier comparison.

**Table 1:** Validation architecture and inferential design of the proposed benchmarking workflow.

Component	Setting
Classification task	Binary (*Inferno* vs *Paradiso*); Ternary (*Inferno*/*Purgatorio*/*Paradiso*)
Preprocessing branches	Leakage free; Deliberately leaky (validation contrast only)
Split structure	Shared splits; Non-shared splits (validation contrast only)
Monte Carlo replicates	*M* = 500 total repetitions (task-specific subsets used depending on the validation contrast)
Train/test proportion	80/20
Internal tuning	5-fold cross-validation
Hyperparameter grid	Regular parameter grid
Statistical comparison	Paired Wilcoxon signed-rank tests; Mann–Whitney U tests
Significance level	α = 0.05
Statistical power	80% target power (pilot assessment for sample size adequacy)

Hyperparameter tuning was performed using regular parameter grids within an internal 5-fold cross-validation. For EN-MNLR, tuning explored the elastic-net mixing parameter together with the regularization penalty over a regular grid spanning the full ridge-to-lasso spectrum and log-scaled penalty values from 10^–6^ to 10^–2^. For SVM, tuning focused on the cost parameter, evaluated over a regular grid spanning log-scaled values from 10^–4^ to 10^2^. Regular grids with 10 levels per parameter were adopted.

### Binary classification

*Inferno* and *Paradiso* are contrasted, representing a relatively easy discrimination problem because strong lexical separation is expected between the two cantiche. This setting functions primarily as a sanity check: both classifiers are anticipated to achieve very high performance, and the main purpose is to verify that the workflow produces stable and consistent estimates under favorable conditions.

Both EN-MNLR and linear SVM achieved near-ceiling performance (Table 2), confirming that the corpus structure provides a stable baseline and that the workflow behaves as expected under conditions of strong class separation. It is worth noting that Table 2 reports performance primarily as a descriptive sanity check using mean ± standard deviation, without implying distributional normality or formal inferential comparison. Summary statistics are computed using the full set of 500 Monte Carlo repetitions. Non-parametric paired inference is reserved for the ternary setting.

**Table 2:** Binary classification sanity-check summary. Values are reported as mean ± standard deviation across 500 Monte Carlo repetitions and are intended as descriptive summaries rather than inferential comparisons.

Leakage	Model	Accuracy	Balanced accuracy	MCC	Macro-F1
Deliberately leaky	EN-MNLR	0.9845 ± 0.035	0.985 ± 0.035	0.972 ± 0.064	0.985 ± 0.035
Deliberately leaky	SVM	0.996 ± 0.018	0.996 ± 0.018	0.992 ± 0.034	0.996 ± 0.019
Leakage-free	EN-MNLR	0.986 ± 0.033	0.986 ± 0.033	0.975 ± 0.063	0.986 ± 0.034
Leakage-free	SVM	0.995 ± 0.020	0.995 ± 0.020	0.991 ± 0.037	0.995 ± 0.020

### Ternary classification and statistical benchmarking

Prior to full repeated validation, it is recommended to use a pilot set of initial Monte Carlo repetitions to estimate the expected effect size and the variability of classifier performance across repeated train/test splits. These preliminary estimates can then be used to approximate the number of repetitions required for adequately powered paired and independent comparisons. In the present study, pilot assessment was based on 100 Monte Carlo repetitions. Since power analysis is usually performed (e.g., using the pwr package in R) under theoretical assumptions that are rarely met in practice, it is prudent to increase the minimum number of repetitions used in statistical benchmarking. In the present study, 100 and 200 repetitions were adopted as conservative operational choices for paired and independent inferential designs, respectively.

Across repeated leakage-free validations, EN-MNLR consistently selected strongly ridge-like solutions, with the elastic-net mixing parameter concentrated near the ridge boundary (median = 0; IQR: 0–0.11). This indicates that predictive stability was generally favored over sparse variable selection. The regularization penalty also remained close to the lower boundary of the explored grid (median = 1 × 10^–6^; IQR: 1 × 10^–6^–2 × 10^–4^), while SVM selected highly stable cost values across repetitions, with the cost parameter consistently centered at 0.22.

**Fig. 1:**
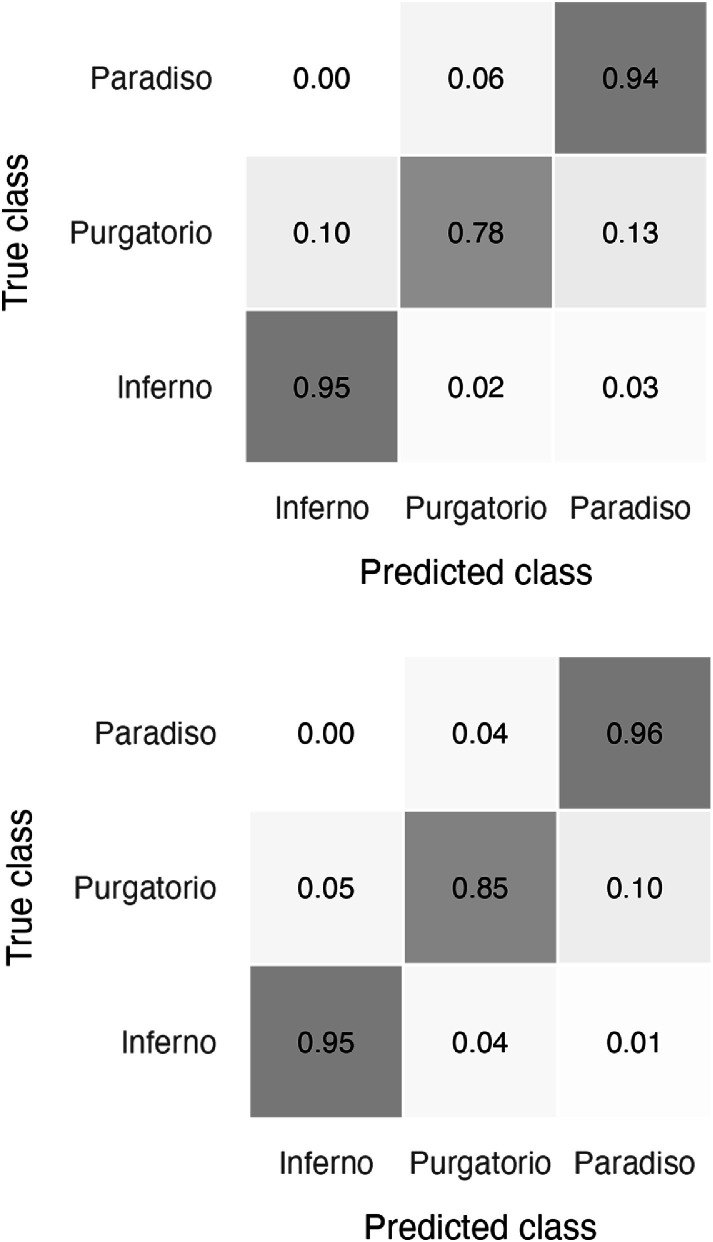
Confusion matrices for EN-MNLR (a) and SVM (b) – ternary leakage-free shared-split design.

SVM showed slightly higher mean performance across all four evaluation metrics (accuracy, balanced accuracy, MCC, and macro-F1), although the magnitude of these differences remained moderate. Confusion matrices for the ternary leakage-free condition provide the clearest summary of classifier behavior (Fig. 1). Misclassifications concentrate primarily around *Purgatorio*, confirming its role as the intermediate and structurally ambiguous class, while direct confusion between *Inferno* and *Paradiso* remains comparatively rare.

On a standard Apple Silicon notebook, a single Monte Carlo iteration required approximately 49 s for EN-MNLR and 13 s for SVM, including internal hyperparameter tuning. Even with repeated internal tuning, the full benchmark remained computationally manageable without requiring parallel execution. The longer computation time observed for EN-MNLR mainly reflects the larger hyperparameter search space and the multinomial regularized fitting procedure, whereas linear SVM required tuning of a single cost parameter only.

### Distribution of performance metrics across repetitions

**Fig. 2:**
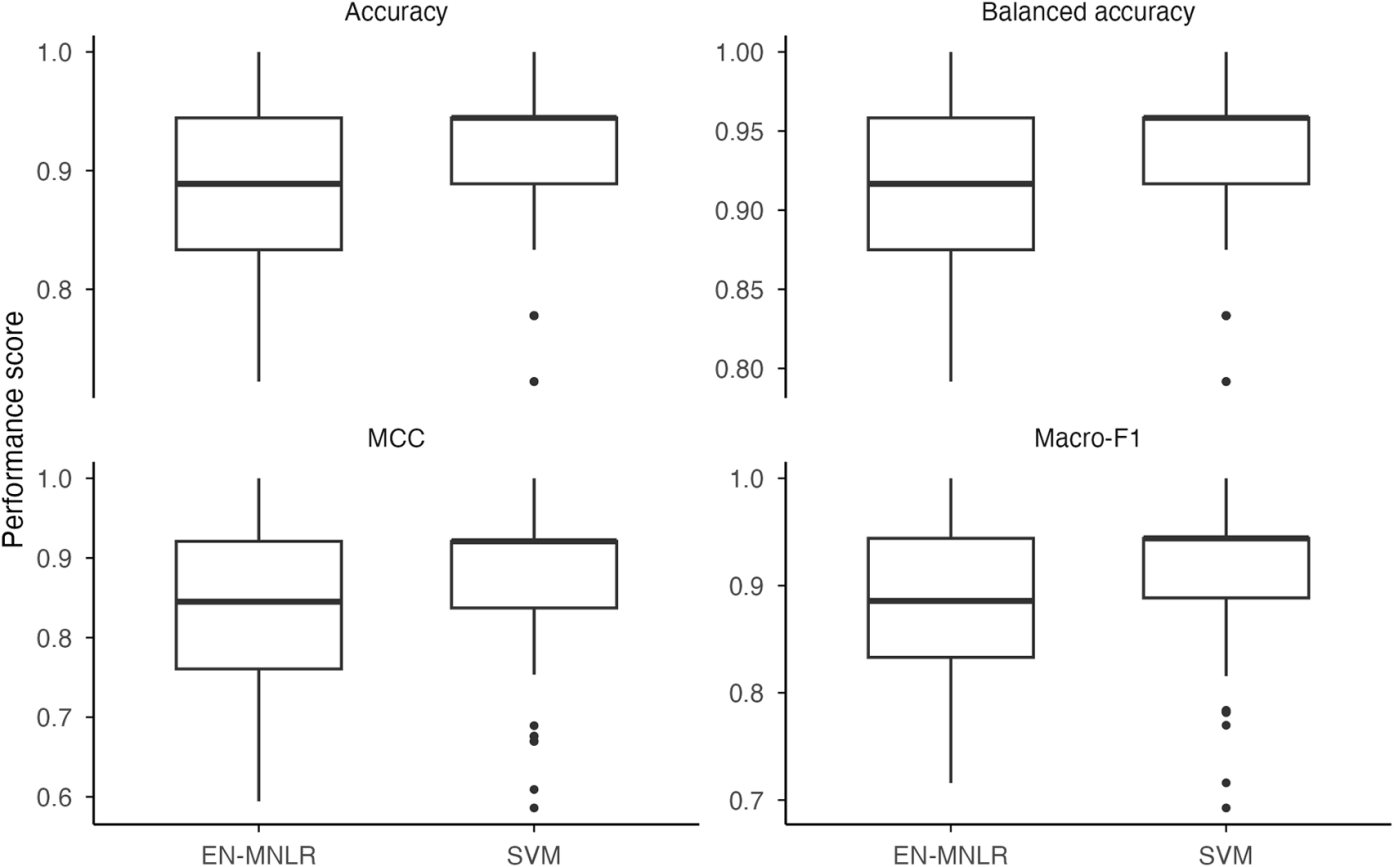
Boxplots of performance scores across 100 Monte Carlo repetitions for EN-MNLR and SVM in the ternary leakage-free setting (shared splits).

To evaluate stability across Monte Carlo repetitions, the distribution of performance metrics was examined using boxplots for the ternary leakage-free condition (Fig. 2). This setting represents the recommended use case of the workflow and therefore serves as the principal basis for the main inferential analysis.

Although both classifiers operated within broadly comparable performance ranges, the repeated-split distributions were clearly non-symmetric and showed substantial left-skewness, particularly for EN-MNLR. Most variability was driven by occasional low-performing repetitions rather than by symmetric dispersion around the median. Linear SVM exhibited a less pronounced left tail, fewer unfavorable realizations, and consistently higher median values, especially for MCC, accuracy and balanced accuracy, indicating greater inferential stability under repeated resampling.

### Paired comparison, independent comparison, and mispaired designs

When both classifiers were evaluated on the same Monte Carlo train/test splits, inferential comparison was performed using paired statistical procedures. This design allows model differences to be evaluated directly within each repetition rather than through independent comparison of summary performance scores.

Under the correct shared-split paired design, SVM showed consistent improvements over EN-MNLR in the ternary task, particularly for MCC, accuracy and balanced accuracy. Paired Wilcoxon signed-rank tests confirmed statistically significant differences across all four evaluation metrics, with moderate effect sizes (Table 3), indicating that the observed advantage was stable across repeated splits rather than driven by isolated high-performing iterations. Effect size for Wilcoxon comparisons was summarized using the rank-based measure $r=\left|Z\right|/\sqrt{N}$. Here, Z is derived from the Wilcoxon test statistic and N is the effective sample size. Following common interpretative conventions, values around 0.1, 0.3, and 0.5 are considered small, small-to-moderate, and large effects, respectively [22].

**Table 3:** Summary of classifier performance comparison for the ternary task under a 100-repetition Monte Carlo design. Shared paired comparisons represent the statistically correct design because both classifiers are evaluated on the same train/test splits. Independent comparisons use non-shared repetitions, while deliberately mispaired comparisons incorrectly treat non-shared repetitions as paired observations.

Leakage-free				
Metric	Shared paired *p*-value	Effect size (*r*)	Independent^†^*p*-value	Mispaired *p*-value
Accuracy	6.32 × 10⁻⁶	0.45	1.18 × 10⁻⁹	1.88 × 10⁻⁴
Balanced accuracy	7.32 × 10⁻⁶	0.45	1.98 × 10⁻⁹	1.56 × 10⁻⁴
MCC	6.26 × 10⁻⁵	0.40	4.77 × 10⁻⁹	1.90 × 10⁻⁴
Macro-F1	1.02 × 10⁻⁶	0.49	2.00 × 10⁻⁹	5.43 × 10⁻⁴

Deliberately leaky				
Metric	Shared paired *p*-value	Effect size (*r*)	Independent^†^*p*-value	Mispaired *p*-value
Accuracy	8.54 × 10⁻⁵	0.39	5.13 × 10⁻⁶	1.86 × 10⁻²
Balanced accuracy	1.90 × 10⁻⁴	0.37	2.73 × 10⁻⁶	1.21 × 10⁻²
MCC	2.27 × 10⁻⁴	0.37	7.08 × 10⁻⁶	3.21 × 10⁻³
Macro-F1	3.74 × 10⁻⁴	0.36	2.78 × 10⁻⁶	4.92 × 10⁻²

Independent comparisons based on non-shared repetitions (200 Monte Carlo repetitions) produced even smaller *p*-values, but this reflects a different inferential question rather than stronger evidence. In that setting, classifier performance is compared across separate Monte Carlo realizations rather than within the same train/test split structure. As shown in the same Table 3, a deliberately mispaired analysis was also performed by treating non-shared repetitions as if they were paired observations. This common but incorrect benchmarking practice produced unstable and potentially misleading conclusions, illustrating that pairing is a property of experimental design rather than a consequence of equal sample size.

The same comparison was repeated under a reduced budget of 25 Monte Carlo repetitions (Table 4). Although effect sizes remained comparable and in some cases even appeared larger, inferential stability deteriorated substantially under deliberately leaky preprocessing, where several mispaired comparisons lost statistical significance entirely. This confirms that small repetition budgets can produce fragile and design-dependent conclusions even when apparent performance differences remain similar.

**Table 4:** Summary of classifier performance comparison for the ternary task under a 25-repetition Monte Carlo design.

Leakage-free				
Metric	Shared paired *p*-value	Effect size (*r*)	Independent†*p*-value	Mispaired *p*-value
Accuracy	1.17 × 10⁻³	0.65	1.88 × 10⁻⁴	1.66 × 10⁻³
Balanced accuracy	6.19 × 10⁻⁴	0.68	1.87 × 10⁻⁴	1.94 × 10⁻³
MCC	1.40 × 10⁻³	0.64	1.82 × 10⁻³	5.36 × 10⁻³
Macro-F1	5.13 × 10⁻⁴	0.69	7.31 × 10⁻⁵	5.53 × 10⁻⁴

Deliberately leaky				
Metric	Shared paired *p*-value	Effect size (*r*)	Independent†*p*-value	Mispaired *p*-value
Accuracy	1.19 × 10⁻²	0.50	7.78 × 10⁻⁴	3.45 × 10⁻¹
Balanced accuracy	9.56 × 10⁻³	0.52	8.63 × 10⁻⁴	2.67 × 10⁻¹
MCC	4.91 × 10⁻³	0.56	1.17 × 10⁻³	3.75 × 10⁻¹
Macro-F1	4.17 × 10⁻³	0.57	7.10 × 10⁻⁴	4.36 × 10⁻¹

† Independent comparisons are based on non-shared train/test splits and therefore require separate Monte Carlo realizations for each classifier. The larger nominal sample size reflects a different inferential design rather than stronger evidence.

Taken together, these results show that reliable classifier comparison depends not only on the choice of statistical test, but also on respecting structural pairing and maintaining a sufficient repetition budget for stable inference.

### Leakage effects and inferential stability

The comparison between leakage-free and deliberately leaky preprocessing showed that leakage is often most problematic precisely when its effect appears small. Average performance changed only modestly across conditions, which could easily suggest that leakage was negligible. However, leakage altered the relative behavior of competing classifiers and changed the stability of inferential comparisons, thereby distorting model selection decisions even in the absence of dramatic accuracy inflation.

**Fig. 3:**
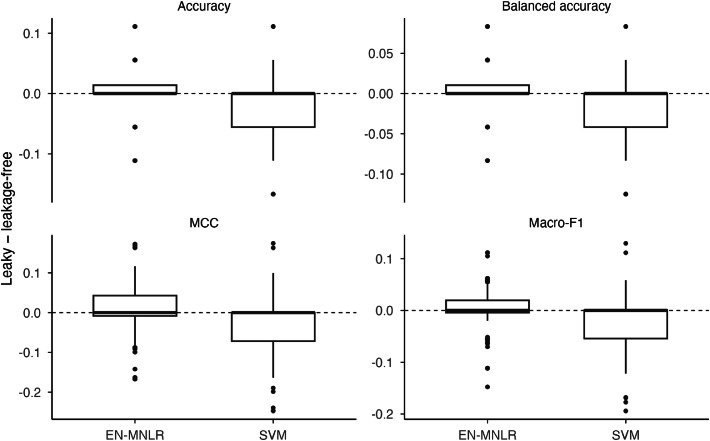
Distribution of paired performance differences between deliberately leaky and leakage-free preprocessing (leaky − leakage-free) across 100 shared Monte Carlo repetitions in the ternary task.

Fig. 3 shows the distribution of paired differences between leaky and leakage-free preprocessing across repeated Monte Carlo splits. The effect was not uniform across models: EN-MNLR remained centered close to zero, with only a small optimistic bias, whereas SVM showed a systematically negative shift, indicating that preprocessing leakage changed the relative ranking and inferential stability of model performance rather than simply inflating all metrics uniformly.

Leakage is particularly insidious: the absence of a large average accuracy gain should not be interpreted as evidence that preprocessing leakage is harmless. This becomes especially relevant when classifiers differ only slightly in predictive performance. In such cases, even a small leakage effect can change the apparent ranking between models and lead to misleading model selection.

This issue is particularly important in literary text analysis, where predictive accuracy is rarely the only objective [14,23]. A classifier that is slightly less accurate but more transparent and interpretable may be preferable to a marginally stronger black-box alternative. In this study, EN-MNLR and SVM provide precisely this contrast.

The purpose of the workflow is therefore not to identify a universally superior classifier, but to support model selection within a design-of-experiments framework, where observed performance remains meaningful under statistically valid evaluation procedures.

### Limitations

Not applicable. The present article describes a methodological workflow for benchmarking supervised classifiers within a design-of-experiments framework rather than proposing a new algorithm or estimator. Methodological scope, modeling choices, and applicability conditions are discussed directly within the Method details and Method validation sections.

## Related research article

Sabatini, A.M. (2026). From graphemic dependence to lexical structure: a Markovian perspective on Dante's *Commedia*. arXiv preprint arXiv:2604.22626 [cs.CL].

## Declaration of competing interest

The authors declare that they have no known competing financial interests or personal relationships that could have appeared to influence the work reported in this paper.

## Data Availability

The complete reproducible workflow is publicly available at https://github.com/angelosabatini/repo_MethodsX and is permanently archived on Zenodo (https://doi.org/10.5281/zenodo.21534964).
